# Retraction: MicroRNA-610 suppresses osteosarcoma oncogenicity via targeting TWIST1 expression

**DOI:** 10.18632/oncotarget.28771

**Published:** 2025-10-06

**Authors:** Chi Jin, Yongjian Feng, Yongjian Ni, Zhonglin Shan

**Affiliations:** ^1^The Third Department of Orthopaedics, Central Hospital of Cangzhou City, Cangzhou, Hebei, China; ^2^The Fourth Department of Orthopaedics, Central Hospital of Cangzhou City, Cangzhou, Hebei, China; ^*^These authors contributed equally to this work

This article has been retracted: Oncotarget’s Image Forensics investigation of this paper has revealed numerous image manipulations, internal and external overlaps and duplications. In particular, the Snail western blot in Figure 3A was duplicated as Twist western blot in Figure 6E and GAPDH image from 3A is duplicated in Figure 6D. The Transwell assay image in Figure 5C is very similar to image in Figure 8I, with some changes introduced by image manipulations. The same Figures 5C, 8I and 8J contain images from Figure 3B of an earlier published paper, which has since been retracted [1]. Images in Figure 5, panels C and D, also were found in Figure 3B of retracted paper [2] and Figure 5C also duplicates an image from Figure 5C of [3], which has also been retracted. The western blot GAPDH image from this paper [3] was also reproduced in Figures 3A and 6D and the same image was found in 2015 papers [4, 5]. The Snail blot from Figure 3A which is identical to the Twist blot from Figure 6E also present in Figure 4J as PIK3CD bands in [6], SP1 bands in [7] and ABCB1 blot in [8]. Therefore, the Editorial decision was made to retract this paper. Oncotarget has attempted to contact the authors several times but has received no replies. All authors either did not respond directly or could not be reached.

Original article: Oncotarget. 2017; 8:56174–56184. 56174-56184. https://doi.org/10.18632/oncotarget.17045
